# GPX3 expression was down-regulated but positively correlated with poor outcome in human cancers

**DOI:** 10.3389/fonc.2023.990551

**Published:** 2023-02-09

**Authors:** Qingyi Hu, Jiaoshun Chen, Wen Yang, Ming Xu, Jun Zhou, Jie Tan, Tao Huang

**Affiliations:** Department of Breast and Thyroid Surgery, Union Hospital, Tongji Medical College, Huazhong University of Science and Technology, Wuhan, China

**Keywords:** GPX3, pan-cancer, glutathione peroxidase, chemotherapy resistance, metastasis, tumor microenvironment (TME)

## Abstract

**Introduction:**

Cancer is a crucial public health problem and one of the leading causes of death worldwide. Previous studies have suggested that GPX3 may be involved in cancer metastasis and chemotherapy resistance. However, how GPX3 affects cancer patients’ outcomes and the underlying mechanism remains unclear.

**Methods:**

Sequencing data and clinical data from TCGA, GTEx, HPA, and CPTAC were used to explore the relationship between GPX3 expression and clinical features. Immunoinfiltration scores were used to assess the relationship between GPX3 and the tumor immune microenvironment. Functional enrichment analysis was used to predict the role of GPX3 in tumors. Gene mutation frequency, methylation level, and histone modification were used to predict the GPX3 expression regulation method. Breast, ovarian, colon, and gastric cancer cells were used to investigate the relationship between GPX3 expression and cancer cell metastasis, proliferation, and chemotherapy sensitivity.

**Results:**

GPX3 is down-regulated in various tumor tissues, and GPX3 expression level can be used as a marker for cancer diagnosis. However, GPX3 expression is associated with higher stage and lymph node metastasis, as well as poorer prognosis. GPX3 is closely related to thyroid function and antioxidant function, and its expression may be regulated by epigenetic inheritance such as methylation modification or histone modification. In vitro experiments, GPX3 expression is associated with cancer cell sensitivity to oxidant and platinum-based chemotherapy and is involved in tumor metastasis in oxidative environments.

**Discussion:**

We explored the relationship between GPX3 and clinical features, immune infiltration characteristics, migration and metastasis, and chemotherapy sensitivities of human cancers. We further investigated the potential genetic and epigenetic regulation of GPX3 in cancer. Our results suggested that GPX3 plays a complicated role in the tumor microenvironment, simultaneously promoting metastasis and chemotherapy resistance in human cancers.

## 1 Introduction

According to the latest worldwide data, crude cancer incidence is still increasing, reflecting its significant socioeconomic burden (1, 2). Lung cancer (LUAD) and breast cancer (BRCA) are the leading malignancies in men and women, respectively. Morbidity and mortality rates for colorectal cancer (COAD), breast cancer, thyroid cancer (THCA), lung cancer, and prostate cancer (PRAD) continue to rise. Cancer metastasis, recurrence, and chemotherapy resistance threaten the life of cancer patients (3). Therefore, we continue to search for new adjuvant therapy and drug combination therapy regimens to enhance the antitumor effect of chemotherapy strategies.

Reactive oxygen species (ROS) and oxidative stress are thought to play several roles in carcinogenesis. For instance, when the gene expression of important molecules governing cell proliferation, apoptosis, or the cell cycle is aberrant, oxidative stress can lead to long-lasting DNA damage and may induce cancer. ROS can promote cancer development by activating multiple signaling pathways (4–9). Therefore, Antioxidants were recommended for cancer prevention and treatment (10–13). Unfortunately, the use of antioxidants in cancer treatment produced disappointing results (14, 15). Antioxidant dietary supplementation has been associated increased incidence and mortality of lung and prostate cancers and promoted breast cancer (16–20). ROS has a dual role in cancer, particularly their contradictory ability to induce cancer cell proliferation or apoptosis (13). In early precancerous and tumor stages, where antioxidant activity was decreased, ROS contribute to cancer progression by generating mutations in oncogenes and tumor suppressor genes (such as RAS and TP53) (4–9). However, as cancer develops into more advanced stages, tumor cells produce large amounts of antioxidants like NADPH and GSH to protect themselves against apoptosis and the associated intratumoral oxidative damage (21, 22). According to previous studies, antioxidants like GSH play a significant role in promoting the emergence and progression of several cancers (23, 24).

Glutathione peroxidase (GPX) is a family of enzymes that protect cells from ROS and play an important role in regulating redox balance (25–28). Glutathione peroxidase 3 (GPX3) located in 5q23 is the only exocrine member of the GPX family and plays an important role in the detoxication of hydrogen peroxide and other oxygen-free radicals (29). GPX3 is expressed in the gastrointestinal, kidney, brain, breast, liver, heart, lung, and adipose tissues (30). Studies have found that serum GPX3 content can be used as a tumor marker (31, 32). GPX3 may be involved in cancer processes by regulating ROS levels. GPX3 is an effective inhibitor of cancer development and progression (33–35). In addition, specific downregulation of GPX3 was found in many types of cancer (36–40). However, GPX3 has also been implicated in metastasis and cancer progression in ovarian, kidney, and thyroid cancers (41–44). It has been reported that the high expression of GPX3 may be associated with abdominal metastasis of serous ovarian adenocarcinoma (41). However, the expression level of GPX3 in tumors has not been extensively studied. The role that GPX3 plays in cancer is unclear. In addition, as a secretory GPX member, the relationship between GPX3 and the tumor microenvironment has not been discussed.

In this study, we explored the role of GPX3 in human tumor diagnosis, prognosis, and sensitivity to treatment and its relationship to the tumor microenvironment.

## 2 Materials and methods

### 2.1 Chemicals, reagents, and antibodies

The Cisplatin (CDDP), Carboplatin (NSC 241240) were purchased from MCE Biological Corporation (CA: HY-17394, NSC 241240). The rabbit normal IgG and antibodies against GPX3 (1:1000, ab256470) was purchased from Abcam (Shanghai, China). Antibodies against GAPDH (1:2000, 60004-1-Ig), HRP-conjugated secondary antibody (1:10000, SA00001-2) were purchased from proteintech (Shanghai, China).

### 2.2 Cell lines, culture conditions, and transduction

MDA-MB-231 and BT-549 are human breast cancer, Lovo and SW480 are human colorectal cancer cell lines, Ovcar-4 is human ovarian cancer, and MKN45 is human gastric cancer cell lines. They were obtained from American Type Culture Collection (ATCC). MDA-MB-231 cultured in L15 (Boster, CA) supplemented with 10% FBS in a humidified atmosphere without CO_2_ at 37°C. BT-549, MKN45 were cultured in RPMI 1640 medium (Gibco, Darmstadt, Germany) supplemented with 10% FBS, Lovo and SW480 was cultured in DMEM (Gibco, Darmstadt, Germany) supplemented with 10% FBS, and they were cultured in a humidified atmosphere of CO2/air (5%/95%) at 37°C. Ovcar-4 was cultured in DMEM/F12 (Gibco, Darmstadt, Germany) supplemented with 10% FBS, and cultured in a humidified atmosphere of CO2/air (5%/95%) at 37°C. We transfected GPX3 knockout adenovirus (shGPX3, 116908-1), GPX3 overexpression adenovirus (oeGPX3, 77869-1) and corresponding control (Ctrl, CON525) into breast cancer (MDA-MB-231, BT-549), colorectal cancer (Lovo, SW480), gastric cancer (MKN45), and ovarian cancer (Ovcar-4) cell lines. All adenovirus were purchased from (Genechem, Shanghai, China).

### 2.3 qRT‐PCR

Total RNA was extracted from the samples with TRIzol (Vazyme, Nanjin, China). In this study, we extracted untreated ovarian cancer (Ovcar-4), breast cancer (MDA-MB-231, BT-549), colorectal cancer (Lovo, SW480) and gastric cancer (MKN45) cells’ RNA to examine the basal expression level of GPX3 in these cells. After transfected GPX3 knockout adenovirus (shGPX3), GPX3 overexpression adenovirus (oeGPX3) and corresponding control (Ctrl) into breast cancer (MDA-MB-231, BT-549), colorectal cancer (Lovo, SW480), gastric cancer (MKN45), and ovarian cancer (Ovcar-4) cells for 72h, their RNA was extracted. Later, we examined the efficiency of adenovirus transfection in regulating GPX3 expression. The complementary DNA was synthesized using a PrimeScript RT reagent Kit (Takara Bio, Otsu, Japan), messenger RNA expression was examined by real-time polymerase chain reaction (RT-PCR) using FastStart Universal SYBR Green Master Mix (Takara Bio, Otsu, Japan) and performed in ABI StepOne Plus Real-time PCR Detection System (Applied Biosystems, Foster City, CA). PCR recycling condition: 95 °C, 5min; 95 °C for 10s, 60 °C for 30s, 40 cycles.

The expression level of GAPDH was simultaneously quantified as an internal standard control. The sequences of all primers (Sangon, Shanghai, China) used were as follows:

GAPDH-F: 5’- TGACATCAAGAAGGTGGTGA-3’
GAPDH-R: 5’- TCCACCACCCTGTTGCTGTA-3’
GPX3-F: 5’- GAGAAGTCGAAGATGGACTGCC-3’
GPX3-R: 5’- AGACCGAATGGTGCAAGCTC-3’

### 2.4 Wound healing assay

The cells were seeded into 6-well-plates. When shGPX3, oeGPX3 and Ctrl cells cover the entire well, we wounded the cells with 200 μL sterile pipette tips. After washing off the floating cells with PBS, the cells were cultured in 1% FBS medium. H_2_O_2_ (5 μM) treated shGPX3, oeGPX3 and Ctrl cells for 4h. After washing off H_2_O_2_ with PBS, the cells were seeded into 6-well-plates. After cells cover the entire well, we performed wound healing procedure as above. The photos were taken under the microscope at 0, 48 hours after injury. Wound Healing size % = (width0 h – width48h)/width0h * 100%.

### 2.5 Transwell assay

5 × 10^5^ cells were seeded into the upper chambers of transwell culture plates (Corning, Shanghai, China). Medium supplemented with 20% FBS (500 μl) was put into the lower chambers. H_2_O_2_ (5 μM) treated shGPX3, oeGPX3 and Ctrl cells for 4h. After washing off H_2_O_2_ with PBS, 5 × 10^5^ cells were seeded into the upper chambers of transwell culture plates. We performed Transwell assay as above. After incubation for 24 h for migration assays, cells penetrated to the lower surface of the membrane and fixed with 4% paraformaldehyde for 60 min and then stained with crystal violet for 30 min and counted.

### 2.6 Clonogenicity assays

For traditional, 5000 cells/well were seeded per well in 6-well plates, and wete cultured for 14 days under normal culture conditions. For cisplatin treatment, cisplatin (dissolved in PBS) was added to cells at clonal density in serum free media for 1 h, cells were then washed twice, and complete growth media was added. In total, 10 - 14 days after seeding plates were fixed with 4% paraformaldehyde for 60 min and then stained with crystal violet for 30 min.

### 2.7 CCK-8 viability assay

The viability of cells seeded in 96-well plates (1000 cells/well) was tested using Cell Counting Kit 8 (CCK-8, (Beyotime, Shanghai, China)). CCK-8 reagent containing serum free media (1:100, 100 µL) was added to each well, and cells were incubated for 1 h. The absorbance was measured at 450 nm using a microplate reader (BioTek, VT).

### 2.8 Drug sensitivity assay

Equal number of cells were seeded into 96-well plates (3000 cells/well) and cell viability in response to different concentration of H_2_O_2_ and cisplatin was measured following 24h after treatment. Cell viability was assessed by using the Cell Counting Kit 8 (CCK-8, (Beyotime, Shanghai, China)) according to the manufacturer’s instructions. The absorbance was measured at 450 nm using a microplate reader (BioTek, VT).

### 2.9 Breast cancer lung metastasis assay in nude mice

Animal experimental procedures were approved by the Ethics Committee of Tongji Medical College, Huazhong University of Science and Technology (IACUC Number: 2612). Athymic nude (nu/nu) mice (4-5 weeks old, female) were purchased from gempharmatech (Jiangsu, China) and fed in a special pathogen-free animal facility and allowed to eat and drink ad libitum. The mice were randomized into 2 groups with 10 mice per group, and then separately inoculated subcutaneously MDA-MB-231/shGPX3 and MDA-MB-231/shCtrl cell suspension. BALB/c nude mice received 2*10^6^ cells (in 100 μL serum-free 1640), directly injected into the tail vein. At the 28 days after injection, lung tissues were harvested, imaged, embedded in 10% paraffin, and subjected to H&E staining.

### 2.10 Bioinformatics analysis

RNA sequencing data and DNA methylation450 data were downloaded from the Cancer Genome Atlas Database (TCGA) (https://portal.gdc.cancer.gov/). Genome-wide GPX3 expression profiles patients were downloaded from TCGA (https://portal.gdc.cancer.gov/). And genetic alteration from TCGA was explored in cBioPortal (https://www.cbioportal.org/). Protein expression of GPX3 was collected from Clinical Proteomic Tumor Analysis Consortium (CPTAC, https://proteomics.cancer.gov/programs/cptac). GPX3 expression in tissue was collected from human protein atlas version 22.0 (HPA, http://www.proteinatlas.org/) (45). The URL links of normal tissues: normal breast tissue (https://www.proteinatlas.org/ENSG00000211445-GPX3/tissue/breast#img), ovarian tissue (https://www.proteinatlas.org/ENSG00000211445-GPX3/tissue/ovary#img), colon tissue (https://www.proteinatlas.org/ENSG00000211445-GPX3/tissue/colon#img), renal tissue (https://www.proteinatlas.org/ENSG00000211445-GPX3/tissue/kidney#img), lung tissue (https://www.proteinatlas.org/ENSG00000211445-GPX3/tissue/lung#img), and endometrium tissue (https://www.proteinatlas.org/ENSG00000211445-GPX3/tissue/endometrium#img). The URL links of cancer tissues: breast cancer (https://www.proteinatlas.org/ENSG00000211445-GPX3/pathology/breast+cancer#img), ovarian cancer (https://www.proteinatlas.org/ENSG00000211445-GPX3/pathology/ovarian+cancer#img), colon cancer (https://www.proteinatlas.org/ENSG00000211445-GPX3/pathology/colorectal+cancer#img), renal cancer (https://www.proteinatlas.org/ENSG00000211445-GPX3/pathology/renal+cancer#img), lung cancer (https://www.proteinatlas.org/ENSG00000211445-GPX3/pathology/lung+cancer#img), and endometrium cancer (https://www.proteinatlas.org/ENSG00000211445-GPX3/pathology/endometrial+cancer#img).GPX3 expression profiles in cell lines were downloaded from Broad Institute Cancer Cell Line Encyclopedia (CCLE, https://portals.broadinstitute.org/ccle/) (46), and drug sensitivity of cancer cell lines were collected from Genomics of Drug Sensitivity in Cancer (GDSC. https://www.cancerrxgene.org/) (47). Median expression was used to dichotomize expression of GPX3, the cutoff to define “high value” at or above the median and below the median define “low value”. Kaplan-Meier curve and receiver operating characteristic (ROC) curve analysis was performed by SPSS 22.0 (SPSS Inc., Chicago, IL, USA). Correlation between drug sensitivity and GPX3 was obtained from TCGA database, Pairwise Pearson correlation between the expression of GPX3 and IC50 of drugs were examined, only a significant correlation (p < 0.05) was retained. DAVID Functional Annotation Bioinformatics Microarray Analysis (https://david.ncifcrf.gov/) was used to perform Gene ontology term enrichment (GO) and Kyoto Encyclopedia of Genes and Genomes (KEGG) pathway analysis. Immune infiltration analysis was performed using CIBERSORT (48), MCPcounter (49), TIMER (50), and xCELL (51) algorithms and online websites.

### 2.11 Statistical analysis

All experiments were performed at least three times. Parametric data are shown as means ± standard deviations (SDs) and nonparametric data as medians and ranges. Two-way ANOVA or one-way ANOVA with Tukey’ s multiple comparison test was used for multiple group analysis. Unpaired Student’ s t-tests were used to compare data between two groups. Two-tailed P-values < 0.05 were considered statistically significant. Statistical analyses were performed using GraphPad prism 9 software (GraphPad software, Inc., La Jolla, CA) and SPSS.

## 3 Results

### 3.1 The expression and correlations of GPX3 in human cancers

**Figure 1:**
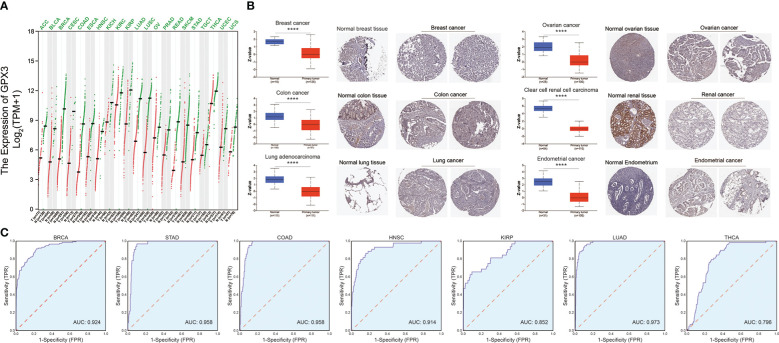
The expression of GPX3 in human cancer. **(A)** The expression of the GPX3 gene in pan-cancer was explored in TCGA and GTEx database. **(B)** Based on the CPTAC analysis and the HPA database, the expression of the GPX3 protein. **(C)** ROC analysis of GPX3 gene in TCGA database.

We compared the expression of GPX3 in human cancers and normal tissues in several public databases. In the TCGA and GTEx databases, we found that GPX3 mRNA expression was downregulated in various types of cancers (**Figure 1A**), including BRCA, COAD, LUAD, ovarian cancer (OV), kidney renal clear cell carcinoma (KIRC), and endometrial cancer (EC). CPTAC analysis and the HPA database demonstrated that the protein expression of GPX3 was also downregulated in BRCA, OV, COAD, ccRCC, LUAD, and EC (**Figure 1B**). ROC curves were used to verify that GPX3 is a valuable diagnostic biomarker in several types of cancers, including BRCA, COAD, LUAD, stomach adenocarcinoma (STAD), head and neck squamous cell carcinoma (HNSC), kidney renal papillary cell carcinoma (KIRP), and THCA, as shown in **Figure 1C** (AUCs > 0.7).

**Figure 2:**
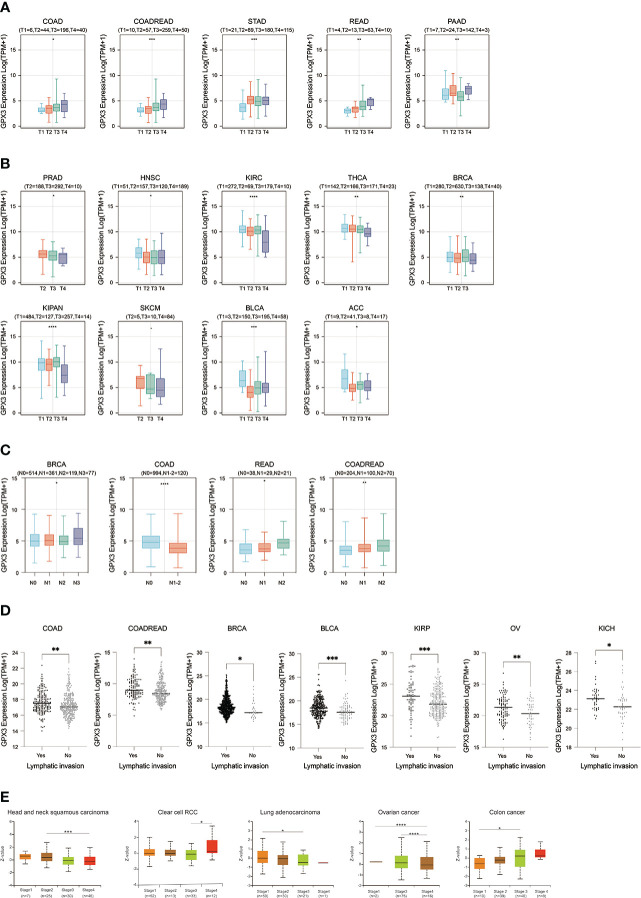
The expression of GPX3 was correlated with stage and lymph node metastases. **(A)** The GPX3 expression was positively correlated with T stage in COAD, READ, STAD, and PAAD. **(B)** The GPX3 expression was negatively correlated with T stage in PRAD, HNSC, KIRC, BRCA, KIPA, SKCM, BLCA, and ACC. **(C)** High GPX3 expression was associated with higher N stage in COAD, READ, BRCA, BLCA, KIRP, OV and KICH. **(D)** High GPX3 expression was associated with lymph node metastases in COAD, READ, BRCA, BLCA, KIRP, OV and KICH. **(E)** GPX3 protein expression was correlated with the pathological stages of HNSC, ccRcc, LUAD, OV, and COAD in CPTAC database.

We used the TCGA database to examine the relationship between GPX3 expression and the pathological stages of human cancer. In COAD, READ, STAD, and PAAD, GPX3 expression was positively correlated with the T stage (**Figure 2A**). However, in PRAD, HNSC, KIRC, BRCA, KIPA, skin cutaneous melanoma (SKCM), bladder urothelial carcinoma (BLCA), and adenoid cystic carcinoma (ACC), GPX3 expression was lower in the higher T stage (**Figure 2B**). In addition, we found in BRCA, COAD, and READ that high GPX3 expression was associated with a higher N stage (**Figure 2C**). We compared the relationship between GPX3 expression level and the presence or absence of lymph node metastasis (**Figure 2D**). In COAD, READ, BRCA, BLCA, KIRP, OV, and KICH, GPX3 expression was increased in the lymph node metastatic group compared with the control group. CPTAC databases showed that GPX3 expression was correlated with the pathological stages of HNSC, ccRcc, LUAD, OV, and COAD (**Figure 2E**).

**Figure 3:**
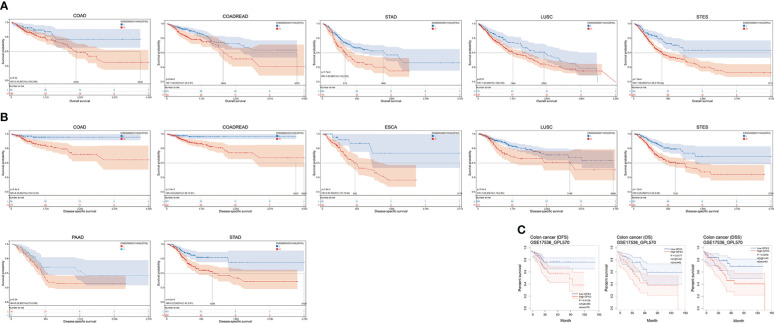
The correlations between GPX3 expression and the prognosis of human cancers. **(A)** GPX3 was negatively associated with OS of COAD, READ, LUSC, STAD, and STES. **(B)** GPX3 was negatively associated with DSS of COAD, READ, ESCA, LUSC, PAAD, STAD, and STES. **(C)** higher GPX3 expression was correlated with poor outcome of COAD patients in GEO database.

The correlations between GPX3 expression and the clinical characteristics of several human cancers, including COAD, prostate adenocarcinoma (PRAD), KIRC, LUAD, and STAD, are shown in **Table 1**. We collected Data from the TCGA database. Patients were divided into a high group and a low group based on the median level of GPX3 expression. Then we compared the differences between the two groups in clinical characteristics. In COAD, GPX3 expression is associated with T-, N-, M-stage, and lymph node metastasis. In PRAD, GPX3 expression is associated with T-, M-stage, lymph node metastasis, and recurrence. GPX3 expression is associated with the M stage in KIRC. In LUAD, GPX3 expression was associated with recurrence. In STAD, GPX3 expression is associated with the M stage and recurrence. Overall, patients with high GPX3 expression and low GPX3 expression in COAD, PRAD, KIRC, LUAD, and STAD showed differences in T-, N-, and M-stage, lymph node metastasis, and occurrence of recurrence. Survival analysis showed that GPX3 expression was associated with the survival of multiple human cancers. High GPX3 expression was associated with poor overall survival (OS) in COAD, READ, LUSC, STAD, and STES (**Figure 3A**) and was associated with poor disease-specific survival (DSS) in COAD, READ, ESCA, LUSC, PAAD, STAD, and STES (**Figure 3B**). The GEO database was also used to show that higher GPX3 expression was correlated with poor outcomes in patients with COAD (**Figure 3C**). Univariate and multivariate Cox analyses were performed to explore the association between GPX3 expression and OS in BRCA, COAD, LUAD, and STAD (**Table 2**). In BRCA, we used univariate analysis to find that risk factors for OS included higher GPX3 expression (p = 0.0170; HR = 1.410), M1 stage (p = 0.009; HR = 1.681), N1-3 stage (p < 0.001; HR = 2.285), T4 stage (p < 0.001; HR = 3.220), and lymph node metastasis (p < 0.001; HR = 2.208). By using multivariate analysis, we found that higher GPX3 expression (p = 0.004; HR = 1.410) and T4 stage (p < 0.001; HR = 2.665) were risk factors for OS. Similarly, in STAD, we used univariate analysis to find that higher GPX3 expression (p = 0.008; HR = 1.533), M1 stage (p = 0.014; HR = 2.052), N1-3 stage (p = 0.004; HR = 1.783), lymph node metastasis (p = 0.002; HR = 1.933) were risk factors for OS. We used multivariate analysis and found that higher GPX3 expression (p = 0.050; HR = 2.428) and the M1 stage (p = 0.037; HR = 2.033) were risk factors for OS. Specific data are shown in **Table 2**. Overall, higher GPX3 expression is a risk factor for OS of BRCA and STAD.

**Table 1:** Relation of GPX3 expression and the clinical characteristic of patients with cancers.

Characteristic	COAD			PRAD			KIRC			LUAD			STAD		
	Low	High	p	Low	High	p	Low	High	p	Low	High	p	Low	High	p
num	209	181		252	299		255	280		307	275		213	194	
**T stage**			**0.009**			**0.007**			0.383			0.625			0.125
T1	9 (3.1%)	2 (0.9%)					126(49%)	149(53%)		99 (32%)	92 (33%)		15 (7%)	7 (4%)	
T2	56 (19.3%)	24(13.0%)		81 (32%)	137 (46%)		35 (14%)	35 (13%)		175 (57%)	146 (53%)		39 (18%)	52 (27%)	
T3	198(68.2%)	121(68.0%)		163(65%)	150 (50%)		86 (34%)	93 (33%)		24 (8%)	26 (9%)		97 (45.5%)	84 (43%)	
T4	24 (11%)	34 (19%)		6 (2%)	7 (2%)		8 (3%)	3 (1%)		8 (3%)	12 (4%)		62 (29%)	43 (22%)	
**N stage**			**0.036**			**0.002**			0.37			0.899			0.452
N0	184(63.4%)	93(54.5%)		171(67.5%)	222(74.2%)		109(43%)	131(47%)		200(65.1%)	171(61.5%)		70 (32.8%)	53 (27.3%)	
N1	62(30%)	45 (25%)		51(20.2%)	29 (9.6%)		10 (4%)	6 (2%)		54 (17.5%)	53 (19%)		52 (24.4%)	56 (28.8%)	
N2-3	42(20.0%)	43 (24.0%)								45 (14.6%)	44(15.7%)		81(39.1%)	73(37.5%)	
**M stage**			**0.045**						**0.009**			0.13			**0.011**
M0	214(73.3%)	129(70.2%)		/	/		188(74%)	236(84%)		197(64.1%)	197(70.8%)		197(92.4%)	161(82.9%)	
M1	33 (16%)	32 (18%)		/	/		45(18%)	33(12%)		13 (4%)	14 (5%)		7 (3.2%)	20 (10.3%)	
**Lymph Node Positive**	99 (47%)	86 (48%)	**0.008**	52 (21%)	29 (10%)	**0.001**	10 (4%)	7 (3%)	0.344				132 (62%)	123 (63%)	0.411
**Relapse, n(%)**	/	/		24 9.5%)	64(21.4%)	**0.008**		5 (1.6%)		94 (30.6%)	67 (24.1%)	**0.046**	20 (9.3%)	42 (21.6%)	**<0.001**

P<0.05, showed bold values.

**Table 2:** Univariate and multivariate analyses of overall survival.

Cancer type (N)	Characteristics	Uni-variate COX analysis		Multivariate COX analysis	
		Hazard ratio (95%CI)	P	Hazard ratio (95%CI)	P
BRCA (1194)	GPX3 (low vs. high)	1.410 (1.063, 1.870)	**0.0170**	1.587 (1.162, 2.167)	**0.004**
	M stage (M0 vs. M1)	1.681 (1.164, 2.426)	**0.009**	0.977 (0.616, 1.547)	0.920
	N stage (N0 vs. N1-3)	2.285 (1.678, 3.110)	**< 0.001**	1.788 (0.777, 4.113)	0.172
	T stage (T1-3 vs. T4)	3.220 (2.019, 5.135)	**< 0.001**	(1.546, 4.594)	**< 0.001**
	lymph node (no vs. yes)	2.208 (1.601, 3.046)	**< 0.001**	1.162 (0.514, 2.627)	0.718
COAD	GPX3 (low vs. high)	1.180 (0.779, 1.786)	0.435	0.861 (0.543, 1.364)	0.523
	M stage (M0 vs. M1)	4.626 (2.931, 7.303)	**< 0.001**	3.023 (1.756, 5.204)	**< 0.001**
	N stage (N0 vs. N1-3)	2.502 (1.639, 3.818)	**< 0.001**	1.413 (0.228, 8.761)	0.710
	T stage (T1-3 vs. T4)	3.377 (2.029, 5.622)	**< 0.001**	2.394 (1.346, 4.258)	**0.003**
	lymph node (no vs. yes)	2.549 (1.650, 3.937)	**< 0.001**	1.248 (0.207, 7.545)	0.809
LUAD	GPX3 (low vs. high)	0.754 (0.574, 0.989)	**0.041**	0.823 (0.603, 1.124)	0.221
	M stage (M0 vs. M1)	2.059 (1.244, 3.410)	**0.005**	1.665 (0.963, 2.880)	0.068
	N stage (N0 vs. N1-3)	2.641 (2.006, 3.479)	**< 0.001**	2.649 (1.933, 3.630)	**< 0.001**
	T stage (T1-3 vs. T4)	1.955 (1.090, 3.507)	**0.041**	1.181 (0.634, 2.201)	0.601
STAD	GPX3 (low vs. high)	1.533 (1.117, 2.106)	**0.008**	2.428 (1.000, 2.040)	**0.050**
	M stage (M0 vs. M1)	2.052 (1.159, 3.632)	**0.014**	2.033 (1.043, 3.962)	**0.037**
	N stage (N0 vs. N1-3)	1.783 (1.206, 2.635)	**0.004**	0.410 (0.048, 3.524)	0.417
	T stage (T1-3 vs. T4)	1.277 (0.897, 1.817)	0.175	1.320 (0.888, 1.962)	0.170
	lymph node (no vs. yes)	1.933 (1.268, 2.946)	**0.002**	4.437 (0.545, 36.089)	0.164

P<0.05, showed bold values.

### 3.2 Intracellular function and regulation of GPX3

**Figure 4:**
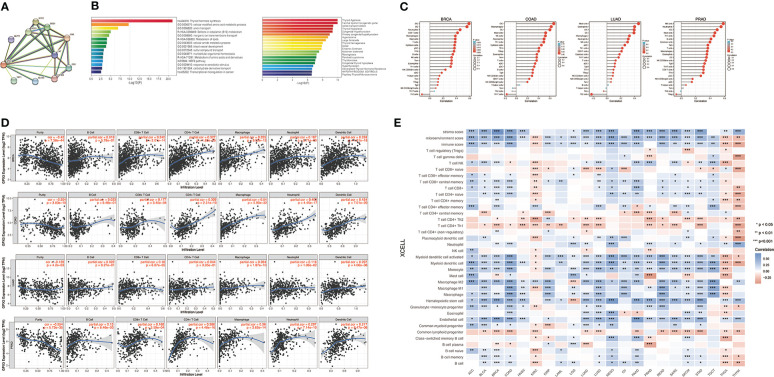
The potential function of GPX3 and effects on immune inflitration in cancers. **(A)** The GPX3-binding PPI analysis. **(B)** The GO and KEGG analysis of GPX3 and GPX3-related partners. **(C)** Correlation analysis between GPX3 expression and immune cells in scatter plots. **(D)** Correlation analysis between GPX3 expression and immune cells in stem-and-leaf display. **(E)** The immune score and cells analysis of GPX3 in pan-cancer.

We used the STRING online database to create a GPX3-binding PPI network (**Figure 4A**) and GO and KEGG analyses (**Figure 4B**) to explore the potential function of GPX3. The results indicated that GPX3 and GPX3-binding proteins were mainly involved in thyroid hormone synthesis and glutathione metabolism.

Furthermore, we explored the relationship between GPX3 and immune invasion in the tumor microenvironment (TME). Through the CIBERSORT, MCPcounter, TIMER, and xCELL algorithms and online websites, we carefully analyzed the relationship between GPX3 expression levels in human cancers and immune score, stromal score, and various cell components in the TME (**Figure 4C–E**; **Table S1**). In most cancer types, the expression level of GPX3 was positively correlated with the stromal score and immune score. Notably, macrophages, especially M2 macrophages, had a consistently positive correlation with GPX3 in various cancers. In addition, immunosuppressive cells in the TME, including myeloid dendritic cells (MDCs) and CD4+ Th1 and Th2 T cells, also had a positive correlation with GPX3. These results suggested that higher GPX3 expression may be related to the immunosuppressive state in the TME.

**Figure 5:**
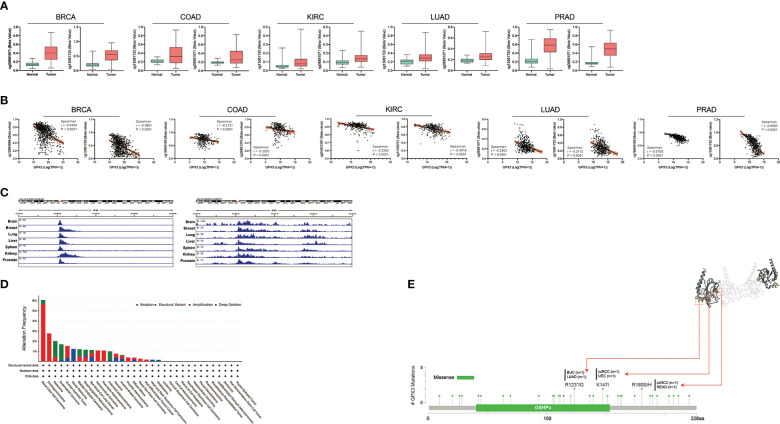
Epigenetic regulation and genetic alteration associated to the expression and structure of GPX3. **(A)** Based on the TCGA database, the DNA methylation level of GPX3 was analyzed in BRCA, COAD, KIRC, LUAD, and PRAD. **(B)** The correlation between GPX3 gene expression and DNA methylation level was analyzed in BRCA, COAD, KIRC, LUAD, and PRAD. **(C)** Enrichment of H3K4me3 and H3K27ac at the upstream of GPX3 in various organs. **(D)** The alteration frequency and mutation type of GPX3. Parts of the mutation sites were displayed within red dotted boxes in the 3D structure of GPX3.

Next, we analyzed the factors regulating GPX3 expression. The epigenetic modification and regulation of GPX3 expression were explored with the Illumina Infinium human methylation 450 and ChIP-Atlas (https://chip-atlas.org) platforms (52, 53). We confirmed that in several cancer types, including lung squamous cell carcinoma (LUSC), PRAD, KIRP, LUAD, BRCA, and COAD, the expression of GPX3 was significantly lower in tumor tissues (**Figure 5A**). Our further analysis showed a negative correlation between GPX3 expression and DNA methylation of the GPX3 promoter region (**Figure 5B**). Enrichment peaks of H3K4me3 and dH3K27ac upstream of GPX3 were also observed in the brain, breast, lung, liver, spleen, kidney, and prostate tissues in our analysis (**Figure 5C**). Taken together, these results indicated that lower expression of GPX3 may be associated with epigenetic factors, including DNA methylation and histone acetylation.

Genetic alteration analysis showed that the overall alteration frequency of GPX3 was > 6%. Missense mutations were found to be the primary type of genetic alteration (**Figure 5D, E**). R123*/G, K147I, and R180S/H were essential alteration sites. They were detected in 1 case of bladder urothelial carcinoma (BUC) and LUAD, 1 case of ccRCC and uterine corpus endometrial carcinoma (UCEC), and 1 case of ccRCC and READ.

### 3.3 GPX3 promotes cancer cell migration

**Figure 6:**
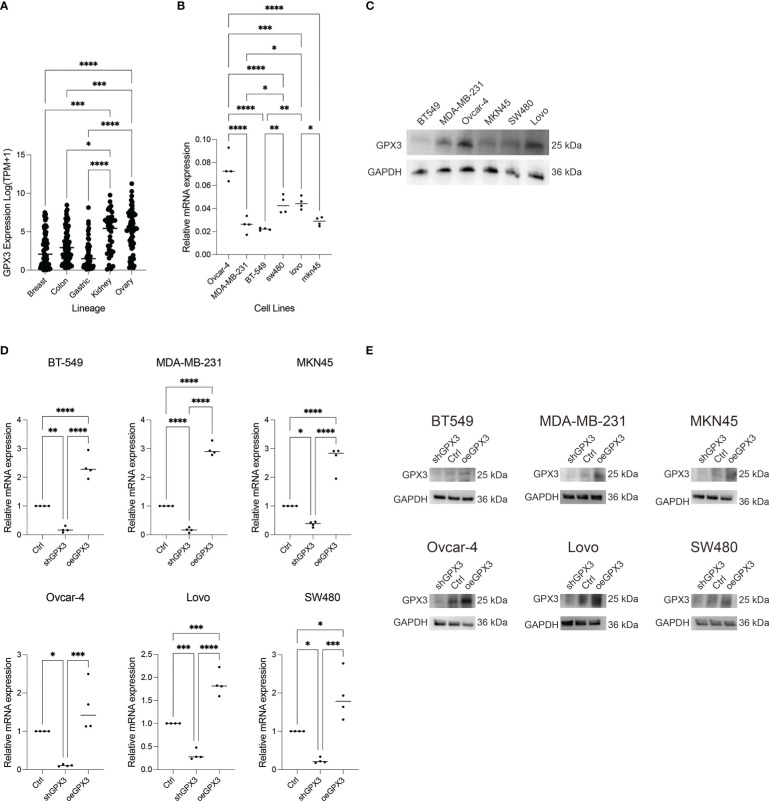
GPX3 expression in ovarian cancer (Ovcar-4), breast cancer (MDA-MB-231, BT-549), colorectal cancer (Lovo, SW480) and gastric cancer (MKN45) cell lines. **(A)** GPX3 expression levels in cancer cells analysed from CCLE database. **(B)** GPX3 expression in cancer cell lines examined by RT-PCR. **(C)** GPX3 expression in cancer cell lines examined by WB. **(D)** GPX3 expression regulation efficiency in cancer cells examined by RT-PCR. **(E)** GPX3 expression regulation efficiency in cancer cells examined by WB. *(p<0.05), **(p<0.01), ***(p<0.001), ****(p<0.0001).

We compared GPX3 expression levels in ovarian cancer, renal clear cell carcinoma, breast cancer, colorectal cancer, and gastric cancer cell lines using the CCLE database. We found that GPX3 expression levels were high in ovarian and renal clear cell carcinoma and moderate in breast, colorectal, and gastric cancers (**Figure 6A**). Then, we used RT-PCR (**Figure 6B**) and WB (**Figure 6C**) to test GPX3 expression levels in ovarian cancer (Ovcar-4), breast cancer (MDA-MB-231, BT-549), colorectal cancer (Lovo, SW480) and gastric cancer (MKN45) cell lines were examined. The results were consistent with CCLE, with the highest expression in ovarian cancer, followed by colorectal cancer and gastric cancer, and moderate expression in breast cancer. We transfected GPX3 knockout adenovirus (shGPX3), GPX3 overexpression adenovirus (oeGPX3), and corresponding control (Ctrl) into breast cancer (MDA-MB-231, BT-549), colorectal cancer (Lovo, SW480), gastric cancer (MKN45), and ovarian cancer (Ovcar-4) cell lines. We used RT-PCR (**Figure 6D**) and WB (**Figure 6E**) to demonstrate the regulatory efficiency of GPX3 expression.

**Figure 7:**
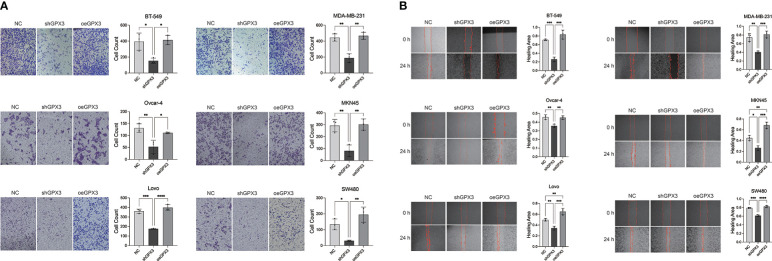
GPX3 promoted human cancer cell migration under oxidation environment. **(A)** Cell migration was assessed 4h following treatment with H_2_O_2_ by using transwell chamber assay. **(B)** Cell migration was assessed 4h following treatment with H_2_O_2_ by by using wound healing assay.

We first examined the effect of GPX3 expression on metastasis. We found that knockdown GPX3 reduced wound healing ability and transmembrane migration ratio of ovarian and colorectal cancer cells (**Figure S1, S2**). But there was no significant effect on the wound healing percentage between shGPX3 and Ctrl of breast and gastric cancer. We used H_2_O_2_ with low concentration to simulate oxidative stress in anoikis during the initial stage of metastasis. After treatment with low concentrations of H_2_O_2_, shGPX3 significantly inhibited the metastasis of cancer cells. In the transwell experiment, compared with the Ctrl, the number of shGPX3 cells decreased significantly under the same magnification field of vision, while the number of oeGPX3 cells did not change compared with the Ctrl (**Figure 7A**). The wound healing experiment was used to compare the change in wound area at the same time. In MDA-MB-231 and BT549 cells, the wound area in the Ctrl group healed 74.4% and 71.0%, respectively, after 48 hours, while that in the shGPX3 group healed only 40.3% and 26.1%, respectively. Similarly, the wound healing area of the shGPX3 group was significantly less than that of the Ctrl group in other cell lines (**Figure 7B**).

Pulmonary metastasis of breast cancer is a manifestation of poor prognosis. We compared the effect of GPX3 on lung metastasis in breast cancer *in vivo*. The MDA-MB-231 cell line bearing shGPX3 showed fewer pulmonary nodules than the NC group *in vivo*. HE staining of lung tissues showed more and larger metastatic cancer cell nests in the Ctrl group (**Figure S3**).

### 3.4 GPX3 showed little effect on proliferation

We first used the CCK-8 assay to compare the effect of GPX3 on the proliferation rate of tumor cells (**Figure S4A**). In ovarian cancer, shGPX3 caused Ovcar-4 proliferation to slow down compared to Ctrl. However, no difference in proliferation rate was observed in the breast, colorectal, or gastric cancer cells. oeGPX3 had no significant effect on the proliferation of these tumor cells.

We observed a different phenomenon in the plate cloning experiment (**Figure S4B**). In ovarian and breast cancer, we observed that the number of clones formed in the shGPX3 group was less than that in Ctrl. However, we found no difference on the number of plate clones between shGPX3 and Ctrl in colorectal or gastric cancer. oeGPX3 also showed no significant influence on the number of clones formed in these cancer cells.

### 3.5 shGPX3 increases oxidative stress damage to cancer cells

**Figure 8:**
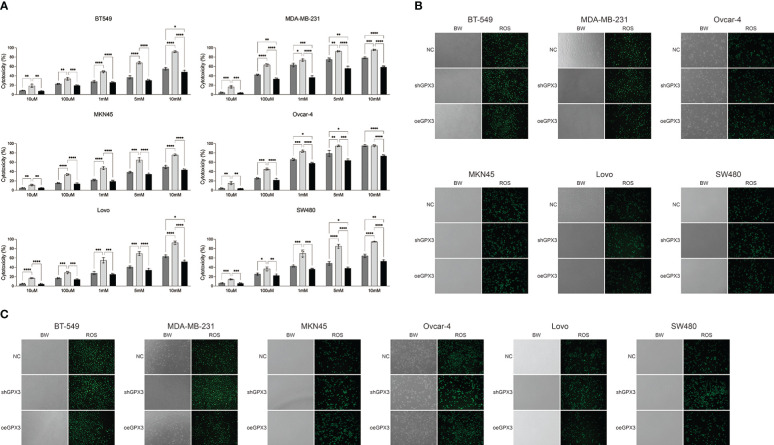
Knockdown GPX3 increased oxidative stress damage in human cancer. **(A)** Cell viability was assessed 24h following treatment with H_2_O_2_ by using cck-8 assay. **(B)** ROS in shGPX3, oeGPX3 and corresponding control cells was assessed by using DCFH-DA probe. **(C)** ROS in cells was assessed 4h following treatment with H_2_O_2_ by using DCFH-DA probe. *(p<0.05), **(p<0.01), ***(p<0.001), ****(p<0.0001).

GPX3 is an important member of the cellular antioxidant system. We examined the effect of GPX3 on cellular oxidative stress resistance. We used a common oxidant, H_2_O_2_, and first compared the sensitivity of several tumor cell lines to H_2_O_2_. We found that downregulated GPX3 caused tumor cells to be more sensitive to oxidants (**Figure 8A**). When a certain concentration of H_2_O_2_ was used to treat tumor cells, shGPX3 resulted in more cell death than the Ctrl, while oeGPX3 partially rescued the loss of cell viability.

We explored the effect of GPX3 on ROS production in tumor cells. We found no significant increase in ROS levels in shGPX3 cells compared with Ctrl cells (**Figure 8B**). We then treated the cells with a lower concentration of H_2_O_2_ and examined intracellular ROS levels. Compared with the Ctrl, oeGPX3 partially reduced intracellular ROS levels, while shGPX3 significantly increased intracellular ROS levels (**Figure 8C**).

### 3.6 shGPX3 increases the sensitivity of tumor cells to platinum-based chemotherapy

**Figure 9:**
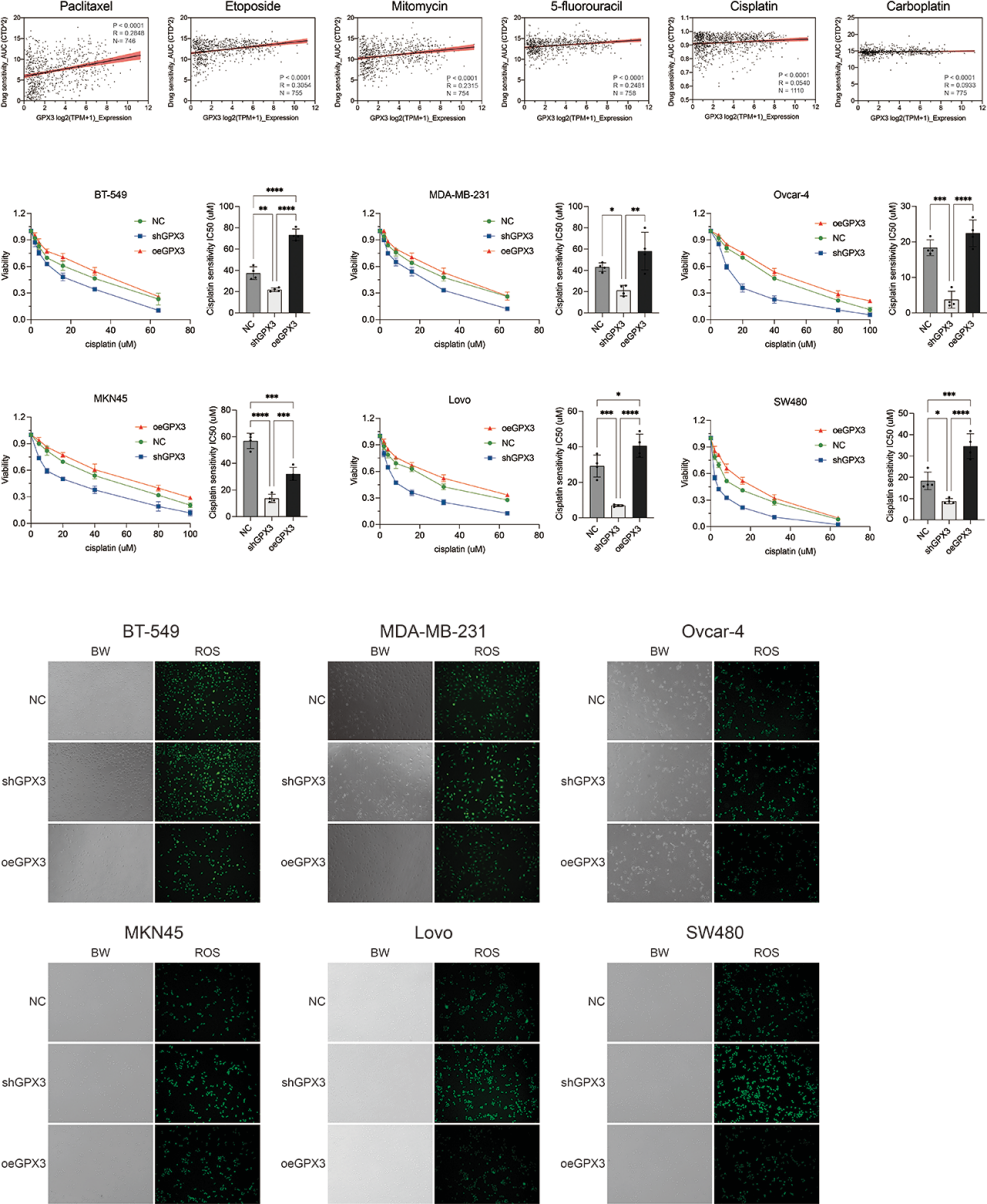
Knockdown GPX3 increased human cancer cell’s sensitivity to cisplatin. **(A)** Expression of GPX3 was positively correlated to IC50 of chemotherapy drugs, including paclitaxel, etoposide, mitomycin, 5-fluorouracil, cisplatin, and carboplatin in pancancer. **(B)** Cell viability was assessed 24h following treatment with cisplatin by using cck-8 assay. **(C)** ROS in cells was assessed 4h following treatment with cisplatin by using DCFH-DA probe. *(p<0.05), **(p<0.01), ***(p<0.001), ****(p<0.0001).

Many chemotherapeutic drugs played antitumor effects by increasing intracellular ROS and causing oxidative stress. Platinum-based chemotherapy, for example, increases intracellular ROS levels and causes large molecules (such as nucleic acids and proteins) damage, ultimately leading to death. We explored the correlation between GPX3 expression and chemotherapy sensitivities in cancer cell lines (54, 55). GPX3 expression data in cancer cells were collected from the cancer cell line encyclopedia (CCLE, https://portals.broadinstitute.org/ccle/) (46) The IC50 drug-sensitive data of cancer cells were collected from genomics of drug sensitivity in cancer (GDSC, https://www.cancerrxgene.org/) (47) (**Figure 9A**). We found that GPX3 expression level was positively correlated with the IC50 of many drugs, including paclitaxel, 5-fluorouracil, carboplatin, etoposide, cisplatin, and mitomycin. Higher GPX3 expression levels were associated with increased IC50 of drugs which means a reduced cell sensitivity to drugs. We speculated that GPX3 played a role in chemotherapy drug resistance in cancers.

We compared the effect of GPX3 on platinum-based chemotherapy sensitivity in several types of cancer cells (**Figure 9B**), and the results showed that shGPX3 resulted in increased sensitivity to platinum-based chemotherapy in breast cancer, ovarian cancer, colorectal cancer, and gastric cancer. We also compared the relationship between ROS level changes induced by cisplatin and GPX3. After the shGPX3 group was treated with cisplatin, the intracellular ROS level increased significantly more than that of the Ctrl and oeGPX3 groups (**Figure 9C**).

## 4 Discussion

GPX3 plays a role in cancer occurrence, progression, and treatment. Our results showed that GPX3 expression was significantly reduced in tumor tissues compared with normal tissues, including BRCA, COAD, HNSC, KIRC, KIRP, LUAD, PRAD, and STAD. The GPX3 expression level had good diagnostic accuracy (AUC>0.75, even 0.9) in BRCA, STAD, COAD, HNSC, KIRP, LUAD, and THCA. In addition, we found that the GPX3 protein expression level was related to the stage. Higher GPX3 expression is significantly associated with higher N-stage in BRCA, COAD, and READ. Compared with primary disease, the expression of GPX3 is higher in metastatic lymph node lesions. These suggested that higher GPX3 expression levels may be associated with early metastasis of human cancers, including COAD, READ, BRCA, BLCA, KIRP, OV, and KICH. For the T-stage, GPX3 played an inconsistent role in different types of cancer, possibly because of the difference in mRNA and protein data from several sources. We investigated the function of GPX3 in the prognosis of cancers. Based on the TCGA and GEO databases, we found that higher GPX3 expression was associated with poor OS in COAD, READ, LUSC, STAD, and STES patients and poor DSS in COAD, READ, ESCA, LUSC, PAAD, STAD, and STES patients. Cui et al. (56) used metabolic-related genes (MRGs) to predict the prognosis of COAD patients. They identified GPX3 as a risk factor in the COAD prognostic model (p < 0.001, HR: 1.006 - 1.023). Khan et al. (57) established the necroptosis-related genes prognostic index (NRGPI). They divided gastric cancer patients into high-risk and low-risk subgroups. The high-risk group showed higher GPX3 expression. Besides, GPX3 was associated with pathways relating to cancer progression and immunosuppression, such as Wnt and TGF-β. GPX3 acted as one of the eight NRGPI oncogenic driver genes and had been validated in gastric cancer cell lines and clinical samples.

Through enrichment analysis of GPX3 and GPX3-related genes, we found that these genes were mainly enriched in thyroid hormone metabolism, glutathione metabolism, and antioxidant activity. This result suggested that GPX3 played an essential role in antioxidant defense. Studies have shown that oxidative stress is a critical metabolic feature of TME inflammatory cell recruitment and may promote the function of tumor-associated fibroblasts (CAFs) (58–60). Malignant tumors can escape immune detection and immunological therapy owing to the development of an immunosuppressive microenvironment. For example, advanced tumors stimulate the formation of an inflammatory immune microenvironment, which inhibits immune-dependent cancer killing. Immune cells secrete cytokines and chemokines, promoting tumor growth, metastasis, and angiogenesis (61–64). Thus, we analyzed the relationship between GPX3 and the tumor immune microenvironment (TIME). The microenvironment of refractory tumors can be divided into immune and inflammatory. In this study, we found that the expression level of GPX3 was positively correlated with immune and stromal scores. We hope GPX3 expression can predict the types of TIME and help select immunotherapy strategies. We found that GPX3 was positively correlated with M2 macrophages, MDCs, CD4+ Th1 cells, Th2 cells, and HSCs. Tumor-associated macrophages (TAMs) in the TME are type M2, which promote angiogenesis and tumor invasion by secreting Th2 cytokines (65). Therefore, GPX3 may be used as a target to rescue immunosuppression in the TME. Notably, the composition of cells in the TME is related to hypoxia. The function of immune cells is impaired by hypoxia and the inflammatory environment. GPX3 plays a role in regulating redox equilibrium, which may be its mechanism in affecting the TIME. M2 macrophages and HSCs are involved in tumor invasion and metastasis (66–70). They are positively related to GPX3 expression. Subsequently, we hoped to explore the relationship between GPX3 expression and tumor metastasis.

In addition, we simply predicted the regulatory mechanism of GPX3 in cancers. We found that there was a significant negative correlation between GPX3 promoter methylation and GPX3 gene expression levels. This suggested that higher DNA methylation in the GPX3 promoter region leads to its lower expression levels in cancer. We also found enrichment peaks of H3K4me3 and H3K27ac in the upstream region of GPX3, suggesting that low GPX3 expression may also be related to histone modification. For genomic variation, the overall alteration frequency of GPX3 was > 6%. Genetic changes may impact the function of GPX3 and further induce malignant transformation and affect the clinical prognosis of cancer patients.

ROS may increase DNA instability, trigger oncogenic mutations and activate oncogenic signaling pathways. Thus, antioxidants may inhibit the initiation or progression of cancer (71). However, in clinical trials, the use of antioxidants did not reduce cancer incidence (72). In contrast, increasing dietary antioxidants increased lung and prostate cancer morbidity and mortality in some studies (16–18). Dietary supplementation with folic acid increases the progression of breast cancer (19, 20). I Antioxidants may promote melanoma metastasis and disease progression in another study (73). It has been reported that glutathione is necessary for the development of some cancers and that antioxidants can promote the development and progression of cancer (74, 75). Clinical studies have shown that compared with benign hyperplasia or precursor lesions, the expression or activity of antioxidant enzymes and GSH content in malignant tumors were increased in the thyroid, ovarian, breast, prostate, and pancreatic cancers (76–81). During carcinogenesis, cells undergo many adaptive changes, especially during metastasis. One such adaptation is that cancer cells enhance their antioxidant defenses to overcome the oxidative stress of anoikis (31). For example, breast and lung cancer cells undergo metabolic changes during metastasis *in vivo* and *in vitro* that reduce ROS production (75, 82–86). In this study, we used multiple human cancer cell lines to examine the effect of GPX3 on metastasis. We found that downregulation of GPX3 expression inhibited metastasis in breast cancer (MDA-MB-231, BT-549), colorectal cancer (Lovo, SW480), gastric cancer (MKN45), and ovarian cancer (Ovcar-4). In terms of proliferation, GPX3 appeared to play a smaller role. Downregulating GPX3 expression slowed the proliferation rate of ovarian cancer (Ovcar-4) and colorectal cancer (Lovo, SW480) cells but did not significantly affect the proliferation of breast cancer or gastric cancer cells. Downregulation of GPX3 significantly inhibited clone formation. shGPX3 significantly reduced the number of clones in ovarian cancer (Ovcar-4), colorectal cancer (Lovo, SW480), and breast cancer (MDA-MB-231). Studies have reported the relationship between the downregulation of GPX3 and tumor metastasis. GPX3 inhibited the migration and invasion of gastric cancer cells (36). However, some studies have found that GPX3 has no antitumor effect in AGS and MKN28 gastric cancer cell lines (87). GPX3 had also been reported to inhibit the progression of breast cancer (35). GPX3 was found to be expressed higher in clear cell type ovarian adenocarcinoma than in other types of ovarian cancer (88). Overall, more studies are needed to determine the role of GPX3 in cancer occurrence, progression, or metastasis. The seemingly contradictory results of GPX3 in cancer may be closely related to ROS. In early cancer and precancer, the expression of GPX3 is decreased and the production of ROS is increased to promote cancer occurrence. However, in advanced cancer, the up-regulation of GPX3 in cancer cells plays a role in eliminating excessive ROS production and protecting cells from anoikis.

GPX3 protected cells from ROS damage in the extracellular environment. We compared the effect of downregulated GPX3 on the antioxidant stress ability of cells. Downregulation of GPX3 expression impairs the antioxidant capacity of cancer cells. Ovarian, breast, colorectal, and gastric cancer cells showed significantly increased sensitivity to oxidants (H_2_O_2_) in shGPX3 compared with Ctrl. In addition, compared to Ctrl, ROS levels in shGPX3 cells were significantly increased after treatment of H_2_O_2_. Knockdown GPX3 significantly decreased the ability of cancer cells to clear ROS. Barrett et al. (34) used the reverse genetics method to study the effect of GPX3 on the occurrence of inflammatory colorectal tumors. GPX3-deficient mouse tumors showed increased inflammation, overactivity of Wnt signaling, and increased DNA damage. Subsequently, they silenced GPX3 in Caco2 making ROS production increase, DNA damage, increased apoptosis in response to H_2_O_2_, and reduced contact-independent growth. Non-contact cell growth is a hallmark of the tumorigenic type. This suggested that acute GPX3 knockdown is indeed detrimental to established cancer growth.

Chemotherapeutic drugs induced ROS accumulation and oxidative stress to produce cytotoxic effects (89). The relationship between the antioxidant capacity of cancer cells and chemotherapy resistance was also frequently reported. By investigating the CCLE and GDSC databases, we found that the expression level of GPX3 was positively correlated with the IC50 of various chemotherapeutic agents. IC50 is commonly used clinically to reflect the sensitivity of cells to drugs. The higher the IC50, the larger the dose of drugs needed to kill cancer cells, thus the lower the sensitivity of cancer cells to chemotherapy. It has been reported that GPX3 was highly expressed in ovarian cancer cells and was associated with platinum resistance (90). Similarly, Pelosof et al. (40) found that decreased GPX3 expression increased the sensitivity of colorectal cancer cell lines to oxaliplatin and cisplatin. Zhou et al. found that GPX3 was the core gene mediating both 5-FU resistance and oxaliplatin resistance in colorectal cancer. They also used tissue chip analysis to determine that patients with high GPX3 expression who received high-intensity chemotherapy regimens (oxaliplatin combination, 6 months of chemotherapy, or 8 cycles of Xeloda) had a significantly increased risk of recurrence and death (91). Platinum is a commonly used chemotherapy drug in the clinic. Pharmacological studies have shown that platinum-induced DNA damage by direct covalent binding with DNA and induced ROS production to destroy protein, DNA, and membrane. GPX3 knockdown resulted in a significant increase in cancer cell sensitivity to platinum-based drugs. In this study, we found that downregulated GPX3 significantly increased the sensitivity of cancer cells to cisplatin, while oeGPX3 promoted the resistance of cancer cells to chemotherapy. Interestingly, we also found that platinum-induced ROS accumulation was most significant in shGPX3, while oeGPX3 eliminated ROS levels.

Overall, pan-cancer analysis of GPX3 illustrated the prospect of GPX3 expression in the prognosis, chemotherapy sensitivity, and immune infiltration of several types of human cancers, providing diagnostic and prognostic biomarkers. Our study further revealed the mechanisms by which GPX3 promotes tumor metastasis, growth, and chemotherapy resistance. There are still many shortcomings in this study. We found a relationship between GPX3 and immune infiltration through bioinformatics analysis. Further experiments are needed to verify how GPX3 affects the tumor immune microenvironment. The specific mechanism by which GPX3 affects cancer susceptibility to chemotherapy also needs further study. In addition, we preliminarily found that GPX3 expression in cancer may be epigenetically regulated, which also needs further verification. In future studies, we will explore these unclear questions in depth.

## Data availability statement

The original contributions presented in the study are included in the article/**Supplementary Material**. Further inquiries can be directed to the corresponding authors.

## Ethics statement

The animal study was reviewed and approved by the Ethics Committee of Tongji Medical College, Huazhong University of Science and Technology (IACUC Number: 2612).

## Author contributions

QH, JC, WY, JT, and TH contributed conception and design of the study. QH, JC, and WY performed experiments and statisitical analysis. QH, JC, MX, and JZ performed the bioinformatics data analysis. QH and WY wrote the first manuscript, QH and JC wrote the revised manuscript. JT and TH edited the language. All authors contributed to the article and approved the submitted version.
