# Supplementary figures and images for: GPX3 expression was down-regulated but positively correlated with poor outcome in human cancers
Source: Front Oncol. 2023 Feb 9;13:990551. doi: 10.3389/fonc.2023.990551 (PMC9947857; doi:10.3389/fonc.2023.990551)

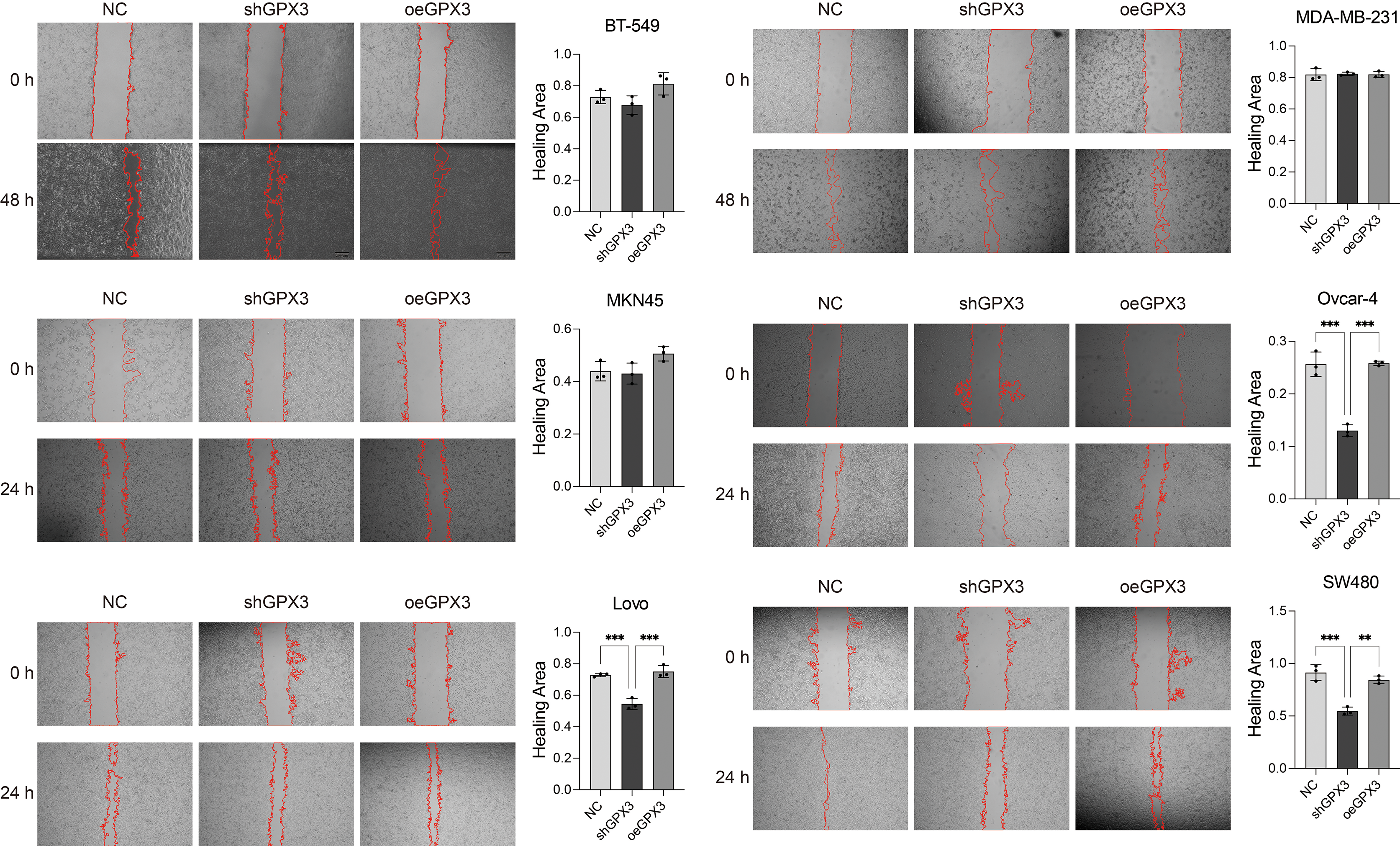

Supplement: Supplementary file 1 [file Image_1.tif]

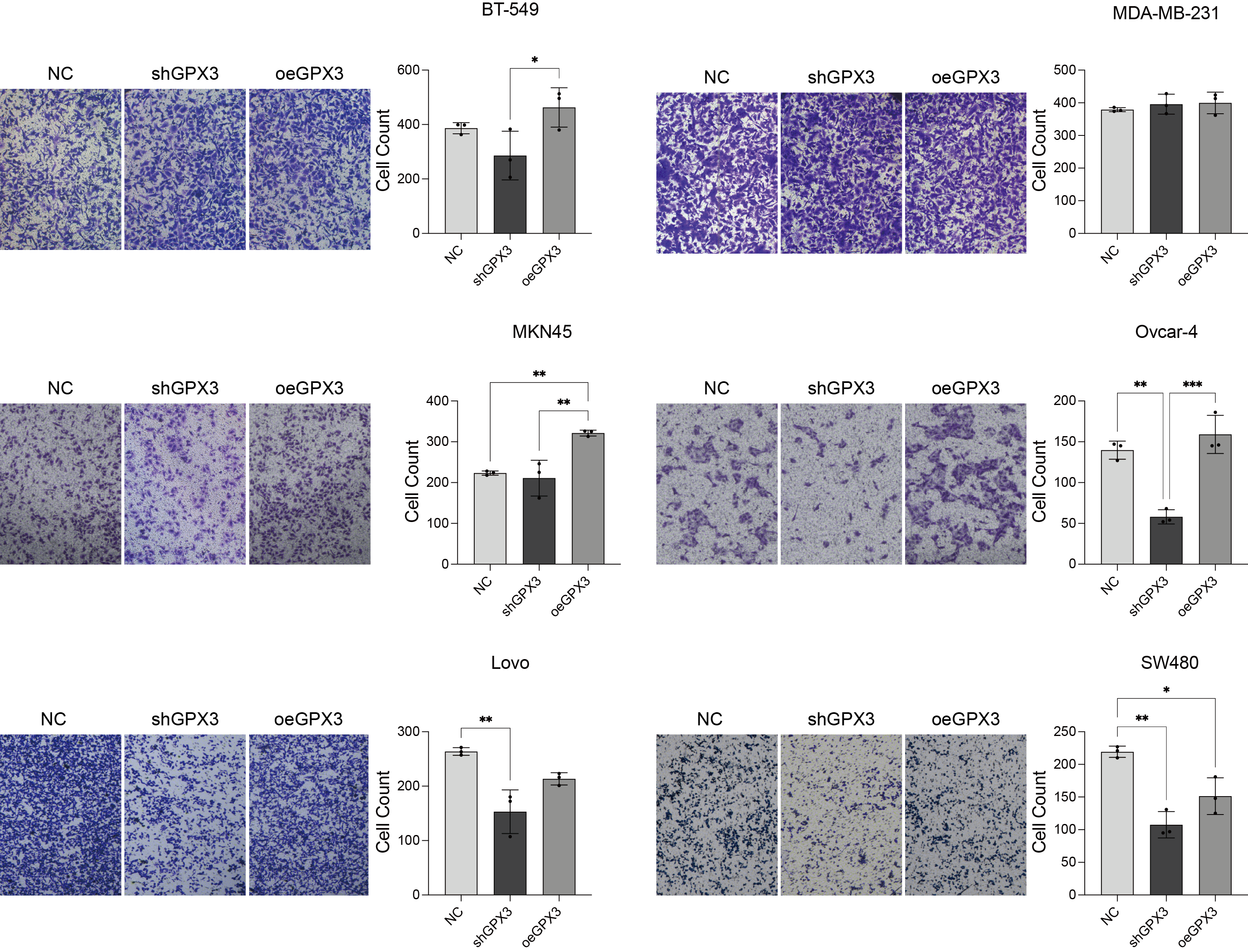

Supplement: Supplementary file 2 [file Image_2.png]

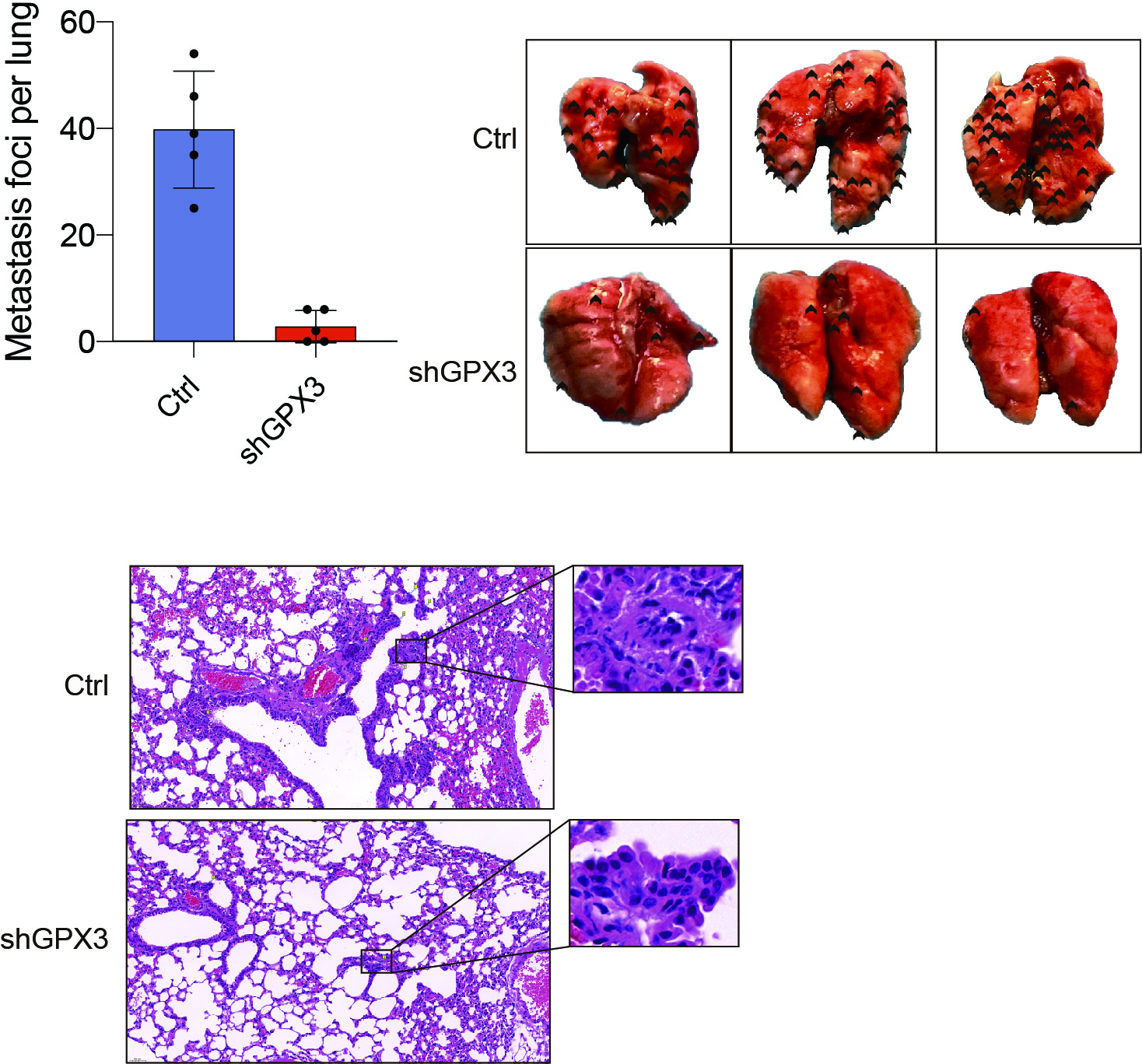

Supplement: Supplementary file 3 [file Image_3.jpeg]

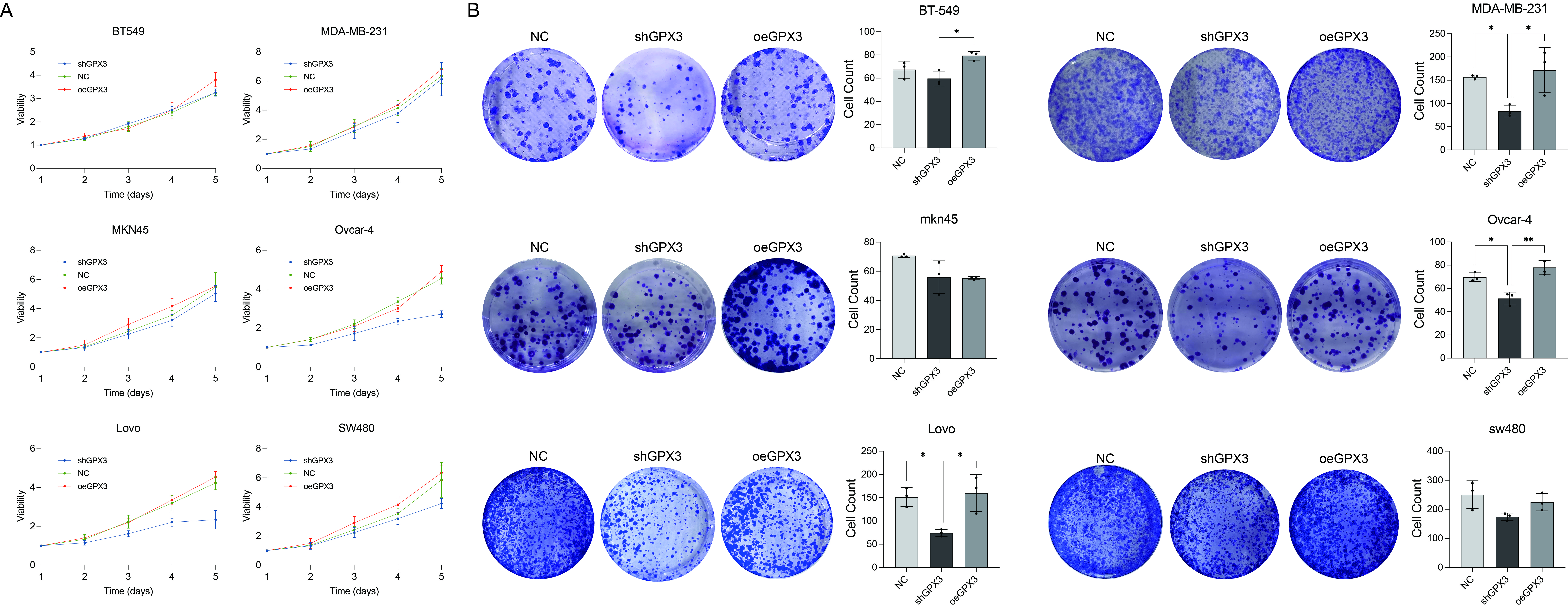

Supplement: Supplementary file 4 [file Image_4.jpeg]
