# Supplementary material for: GPX3 expression was down-regulated but positively correlated with poor outcome in human cancers
Source: Front Oncol. 2023 Feb 9;13:990551. doi: 10.3389/fonc.2023.990551 (PMC9947857; doi:10.3389/fonc.2023.990551)
Supplement: Supplementary file 5 [file Table_1.docx]

**Table S1**. Correlation analysis between GPX3 expression and immune cells.

| **Variable** | **BRCA** | | **COAD** | | **LUAD** | | **PRAD** | |
| --- | --- | --- | --- | --- | --- | --- | --- | --- |
|  | **Cor** | **P** | **Cor** | **P** | **Cor** | **P** | **Cor** | **P** |
| Purity | -0.420 | <0.001 | -0.304 | <0.001 | -0.129 | <0.001 | -0.554 | <0.001 |
| B Cell | 0.018 | 0.578 | 0.023 | 0.648 | 0.022 | 0.627 | 0.130 | <0.001 |
| CD8+ T Cell | 0.242 | <0.001 | 0.177 | <0.001 | 0.180 | <0.001 | 0.168 | <0.001 |
| CD4+ T Cell | 0.327 | <0.001 | 0.309 | <0.001 | 0.044 | 0.335 | 0.386 | <0.001 |
| Macrophage | 0.225 | <0.001 | 0.540 | <0.001 | 0.284 | <0.001 | 0.360 | <0.001 |
| Neutrophil | 0.187 | <0.001 | 0.403 | <0.001 | 0.116 | 0.011 | 0.297 | <0.001 |
| Dendritic Cell | 0.259 | <0.001 | 0.434 | <0.001 | 0.207 | <0.001 | 0.217 | <0.001 |
